# Reviews and Book Notices

**Published:** 1880-10

**Authors:** 


### Reviews and Book Notices.

Article VII.—Neuralgia: Its Nature and Curative Treatment. By Thomas Stretch Dowse, m.d., f.r.c.p., Edin. London: Bailliere, Tindall & Cox; 1880.

This work is offered as the second volume of a series on "Diseases of the Brain and Nerves," the first volume of which, on "Syphilis of the Brain," appeared during the past year and has, we believe, been republished in this country. Its inclusion in this series seems to have been an afterthought of the author, who in a manner apologizes for the fact. As regards the scope and style of the work, he says: "If the reader expects to find, in the following pages, any vague and speculative theories relative to the pathology of neuralgia, he will be greatly mistaken. Yet the author hopes that the reader will find therein the great question relative to the practical and curative treatment of neuralgia discussed in a simple, comprehensive and concise manner."

His ideas on the pathology of the disease are, however, vague enough if not speculative; they seem to be based on Romberg's epigrammatic saying that it is "the prayer of the nerve for healthy blood," a statement altogether more rhetorical than scientific, and one which, however captivating it may sound, is a very inadequate expression of the true facts of the pathology of neuralgia. Our author defines neuralgia as "a diseased condition of the blood and tissues of the body, and a condition which causes a more or less definite and localized painful affection of the nerves." This is certainly vague and inaccurate enough, and we do not find, in the succeeding paragraphs, any good evidence of very clear ideas on the real conditions in neuralgia. The author's disclaimer, therefore, of any special claims as a theoretical pathologist is well supported, and his characteristics in this respect are noticeable throughout the book.

On the practical side of neuralgia the author is much more satisfactory. He follows a very elaborate classification of the forms of neuralgia, giving illustrative cases under each head, with some generally very sensible and practical remarks on each variety. We notice here, however, what is perhaps an excess of faith in the treatment employed, nearly every clinical history being headed " cured by " this or that remedy. His treatment is also in some particulars what would be deemed in this country heroic ; thus he advises one-fourth or one-half of a grain of morphia with one-twentieth or one-fortieth of a grain of atropia for a hypodermic injection, and one-half to one grain of morphia for a single beginning dose internally, without any caution or specification as to idiosyncracy or previous accustomation to the drug. But the reader will find this part of the book—and it really amounts to about the whole of it—full of useful, practical suggestions, some of which may be novel. The work is on this account well worthy of perusal and we recommend it, not as supplanting, but as supplementary to other more scientific treatises on its subject. H. M. B.

ARTICLE VIII.—A MANUAL OF SURGERY. By T. FAIRLIE CLARKE, Assistant Surgeon to Charing Cross Hospital. Wm. Wood & Co., 1879.

This, as the title implies, is a handbook for the general practitioner, and contains a little of everything pertaining to surgery.

The book is thoroughly revised and improved by an American Surgeon, whose additions here and there bring the work fully up to date.

The additions are inclosed in brackets and consist mostly of familiar subjects to the students of the past few years; such as Esmarch's bandages, Sayre's plaster of Paris jacket, Buck's apparatus for fracture of the thigh, Sayre's dressing for fractured clavicle, Lister's antiseptic method ; Bigelow's litholapaxy, closing with a whole added chapter on transfusion.

This closes the series of works comprised in Wood's Medical Library for 1879.

The whole set fulfill the promises which heralded them and are really marvels of cheapness and excellence. The binding is of uniform taste and strength and looks well on the shelves. Several of the numbers are classical in excellence, and have passed through several French and German editions. They have been received by the profession, and reflect great credit upon the judgment and enterprise of the publishers. W. L. D.

ARTICLE IX.—OFFICIAL REGISTER OF PHYSICIANS AND MIDWIVES TO WHOM CERTIFICATES HAVE BEEN ISSUED BY THE ILLINOIS STATE BOARD OF HEALTH—12 mo. Weber & Co,, Springfield, State Printers, 1880.

The Register reflects great credit on the efficient Secretary (Dr. J. H. Rauch) of the State Board of Health. It contains a copy of the Act to Regulate the Practice of Medicine in the State of Illinois, and a copy of the Act to Create and Establish a Board of Health in this Commonwealth, and a Register of Physicians, Regular, Homœopathic, Eclectic, Botanic, Physico-Medical, Thompsonian, and Midwives, arranged by counties, giving the date of registration, school, residence, age, nativity, total years of practice, etc., of each physician arranged by counties and alphabetically. We have examined the Register carefully so far as the regulars are concerned, and find it a marvel of accuracy.

The State Board of Health certainly deserve credit for the thoroughness with which they do their work, and we hope the State legislature will see proper to enlarge their powers so that they may succeed in the laudable undertaking of elevating and purifying the medical profession. D. R. B.

ARTICLE X.—A TREATISE ON FOREIGN BODIES IN SURGICAL PRACTICE. By Alfred Poulett, M.D., Inspector of the School for Military Medicine at Val-de-Grace. 2 Vols. Wm. Wood & Co., 1880.

This unique work contains a vast amount of information on a subject hitherto unnoticed in this form. The author has collected from every source, chiefly from the large number of cases reported hitherto in medical journals from all parts of the world, his own ample experience and that of his colleagues, cases of all kinds of foreign bodies in every cavity of the body, their diagnosis, prognosis, and treatment. In the description of each body and its location, the character of the secretion is considered, the viscus in which it is lodged, and its influence upon the intruder. Some of the well-authenticated cases he details, especially of foreign bodies, in the urinary and genital tract are almost incredible. The author's dry comments upon them are amusing. A more novel and interesting work seldom appears.

ARTICLE XI.—A CLINICAL TREATISE ON DISEASES OF THE NERVOUS SYSTEM. By M. ROSENTHAL, Professor of Diseases of the Nervous System at Vienna. With a preface by PROFESSOR CHARCOT of Paris. New York: Wm. Wood & Co.; 1879.

This classical work has run through three large German and French editions since 1870, and has met with extremely favorable criticism everywhere. It is exhaustive and thorough, especially in the practical departments of symptomatology and therapeutics. In this respect it is especially adapted to the wants of the general practitioner. The author uses hydropathic treatment in many chronic nervous diseases in simple and moderate methods, and pays much attention to medical electricity both for diagnosis and treatment. The work is translated by Dr. M. Putzel of New York, who has introduced a number of very serviceable wood-cuts at appropriate places. The two volumes are included in Wm. Wood & Co.'s Cheap Series for 1879.

ARTICLE XII.—MATERIA MEDICA AND THERAPEUTICS—VEGETABLE KINGDOM. By CHARLES D. F. PHILLIPS, M.D., F.R.C.S.E., Lecturer on Materia Medica, Westminster Hospital. New York: Wm. Wood & Co.; 1879.

Another reprinted English work edited by an American author, the well known Henry G. Pifford of New York City, who has adapted it to the American pharmacopœa and sufficiently condensed it to bring it within the limits of the series in which it appears. A few unimportant articles are omitted to make room for newer and more useful drugs, as jaborandi, gelseminum, iris versicolor, etc. Both apothecaries' and metric weights are given, the latter in brackets. The work is indexed, both as to remedies and diseases. The second portion, devoted to drugs of inorganic action, is nearly ready for the press. W. L. D.

ARTICLE XIII.—THE SURGERY, SURGICAL PATHOLOGY AND SURGICAL ANATOMY OF THE PELVIC ORGANS. With commentaries, notes, and cases. By Henry Savage, M.D., London, etc. Wm. Wood & Co., Standard Medical Library, 1880.

This work of only 129 pages, is profusely illustrated by thirty-two excellent plates, each containing from four to eight different illustrations, supplemented by thirty-two wood cuts giving diagrams of the pelvis and its contents in every possible section, with minute dissections of each tissue in its anatomical and surgical relations. The operations for ruptured perineum and ovariotomy are graphically illustrated in each stage. In the latter the author has frequently assisted Mr. Wells, the celebrated ovariotomist, and is a colleague of that gentleman. While some of the author's theories, in the glimpses he gives us of them, may be peculiar, still the book is admirable in just what the title announces, the Surgical and Pathological Anatomy of the Pelvis.

ARTICLE XIV.—INFANT FEEDING AND ITS INFLUENCE ON LIFE; OR, THE CAUSES AND PREVENTIONS OF INFANT MORTALITY. By C. H. F. ROUTH, M.D., M.R.C., P.L.

The author gives the result of many years of painstaking observation. He is evidently in love with his work; and there is not a neglected detail or a question one might ask but is answered. The book reads easily, is well indexed, and provided with an appendix full of English and French statistics of infantile mortality.

ARTICLE XV.—DISEASES OF THE INTESTINES AND PERITONEUM, comprises a collection of monographs on enteralgia, enteritis, obstruction of the bowels, ulceration of the bowels, cancerous and other growths of the intestines, diseases of the cæcum and appendix vermiformis, colic, colitis and dysentery, diseases of the rectum and anus, intestinal worms, peritonitis, tubercle of the peritoneum, carcinoma of the peritoneum, affections of the abdominal lymphatic glands, and ascites, by a number of noted

English writers, form also a volume of 247 pages in Wood's Medical Library. The articles, for the most part, are terse and interesting, though one or two are in rather old style. No practitioner will be disappointed with the time spent over the book.

